# Medication-Related Adverse Events and Discordancies in Cystatin C–Based vs Serum Creatinine–Based Estimated Glomerular Filtration Rate in Patients With Cancer

**DOI:** 10.1001/jamanetworkopen.2023.21715

**Published:** 2023-07-05

**Authors:** Paul E. Hanna, Qiyu Wang, Ian A. Strohbehn, Daiana Moreno, Destiny Harden, Tianqi Ouyang, Nurit Katz-Agranov, Harish Seethapathy, Kerry L. Reynolds, Shruti Gupta, David E. Leaf, Meghan E. Sise

**Affiliations:** 1 Division of Nephrology, Department of Medicine, Massachusetts General Hospital, Boston; 2 Division of Hematology and Oncology, Department of Medicine, Massachusetts General Hospital, Boston; 3 Division of Renal Medicine, Brigham and Women's Hospital, Boston, Massachusetts; 4 Adult Survivorship Program, Dana-Farber Cancer Institute, Boston, Massachusetts

## Abstract

**Importance:**

Serum creatinine–based estimated glomerular filtration rate (eGFRcr) may overestimate the glomerular filtration rate (GFR) in patients with cancer. Cystatin C–based eGFR (eGFRcys) is an alternative marker of GFR.

**Objective:**

To determine whether the therapeutic drug levels and adverse events (AEs) associated with renally cleared medications were higher in patients with cancer whose eGFRcys was more than 30% lower than their eGFRcr.

**Design, Setting, and Participants:**

This cohort study analyzed adult patients with cancer at 2 major academic cancer centers in Boston, Massachusetts. These patients had their creatinine and cystatin C measured on the same day between May 2010 and January 2022. The date of the first simultaneous eGFRcr and eGFRcys measurement was considered to be the baseline date.

**Exposure:**

The primary exposure was eGFR discordance, defined as an eGFRcys that was more than 30% lower than the eGFRcr.

**Main Outcomes and Measures:**

The primary outcome was risk of the following medication-related AEs within 90 days of the baseline date: (1) supratherapeutic vancomycin trough level greater than 30 μg/mL, (2) trimethoprim-sulfamethoxazole–related hyperkalemia (>5.5 mEq/L), (3) baclofen toxic effect, and (4) supratherapeutic digoxin level (>2.0 ng/mL). For the secondary outcome, a multivariable Cox proportional hazards regression model was used to compare 30-day survival of those with vs without eGFR discordance.

**Results:**

A total of 1869 adult patients with cancer (mean [SD] age, 66 [14] years; 948 males [51%]) had simultaneous eGFRcys and eGFRcr measurement. There were 543 patients (29%) with an eGFRcys that was more than 30% lower than their eGFRcr. Patients with an eGFRcys that was more than 30% lower than their eGFRcr were more likely to experience medication-related AEs compared with patients with concordant eGFRs (defined as eGFRcys within 30% of eGFRcr), including vancomycin levels greater than 30 μg/mL (43 of 179 [24%] vs 7 of 77 [9%]; *P* = .01), trimethoprim-sulfamethoxazole–related hyperkalemia (29 of 129 [22%] vs 11 of 92 [12%]; *P* = .07), baclofen toxic effects (5 of 19 [26%] vs 0 of 11; *P* = .19), and supratherapeutic digoxin levels (7 of 24 [29%] vs 0 of 10; *P* = .08). The adjusted odds ratio for vancomycin levels more than 30 μg/mL was 2.59 (95% CI, 1.08-7.03; *P* = .04). Patients with an eGFRcys more than 30% lower than their eGFRcr had an increased 30-day mortality (adjusted hazard ratio, 1.98; 95% CI, 1.26-3.11; *P* = .003).

**Conclusions and relevance:**

Results of this study suggest that among patients with cancer with simultaneous assessment of eGFRcys and eGFRcr, supratherapeutic drug levels and medication-related AEs occurred more commonly in those with an eGFRcys more than 30% lower than their eGFRcr. Future prospective studies are needed to improve and personalize GFR estimation and medication dosing in patients with cancer.

## Key Points

**Question:**

What are the consequences of discordance in serum creatinine–based estimated glomerular filtration rate (eGFRcr) vs cystatin C–based eGFR (eGFRcys) in patients with cancer?

**Findings:**

In this cohort study of 1869 adults with cancer, those with an eGFRcys that was more than 30% lower than their eGFRcr had an increased risk of supratherapeutic vancomycin levels, trimethoprim-sulfamethoxazole–related hyperkalemia, baclofen toxic effect, high digoxin levels, and increased risk of death within 30 days.

**Meaning:**

Findings of this study suggest that medication-related adverse events occurred more commonly in patients whose eGFRcys was more than 30% lower than their eGFRcr, necessitating future studies to improve and personalize glomerular filtration rate estimation and medication dosing in patients with cancer.

## Teaser

This cohort study assesses the risk of supratherapeutic doses, hyperkalemia, toxic effects, and mortality among adults with cancer with lower serum creatinine–based than cystatin C–based estimated glomerular filtration rate.

## Introduction

Accurate assessment of estimated glomerular filtration rate (eGFR) is key to dosing renally cleared medications. While the criterion standard method for evaluating glomerular filtration rate (GFR) is direct measurement (measured GFR [mGFR]) using exogenous filtration markers (eg, iohexol),^1^ GFR estimation using serum creatinine (SCr) is the most commonly used method in both clinical practice and research.^2,3,4^ Creatinine is a byproduct of muscle metabolism that is filtered and secreted by the kidneys. Despite an increase in precision of currently available eGFR equations, SCr-based eGFR (eGFRcr) remains inaccurate and can overestimate GFR, particularly in patients with sarcopenia.^5,6^ This overestimation can lead to inaccurate dosing of medications that require adjustment based on eGFR, such as commonly used antibiotics, muscle relaxants, antiepileptic drugs, blood thinners, and antiarrhythmic medications.

Cystatin C is a low-molecular-weight (13 000 Da) protein produced by all nucleated cells that has been increasingly used as an alternative to SCr to estimate GFR.^2,5,7^ Cystatin C is not readily affected by age, sex, muscle mass, or diet; however, there are non-GFR factors associated with cystatin C. It is elevated in acute inflammation, obesity, and thyroid disease and with corticosteroid use.^8,9,10,11^ A recent large study in patients with solid tumors found that SCr overestimated GFR, cystatin C underestimated GFR, and an equation that combines both SCr and cystatin C was the most accurate and precise way to estimate GFR.^12,13^

Because cancer is an important risk factor for sarcopenia and frailty,^14^ we hypothesized that having a cystatin C–based eGFR (eGFRcys) that is substantially lower than an eGFRcr would be common in patients with cancer. Few studies have evaluated the implications of discordance between eGFRcr and eGFRcys for the clinical outcomes associated with medication use. Given that patients with cancer are commonly exposed to numerous medications that require dose adjustment by eGFR, we sought to determine whether the therapeutic drug levels and adverse events (AEs) associated with renally cleared medications were higher in patients with cancer whose eGFRcys was more than 30% lower than their eGFRcr.

## Methods

The Mass General Brigham Institutional Review Board approved this cohort study and waived the informed consent requirement because retrospective data were used. We followed the Strengthening the Reporting of Observational Studies in Epidemiology (STROBE) reporting guideline.

### Patient Population and Data Collection

Using the Research Patient Data Registry, Mass General Brigham’s centralized data warehouse,^15,16^ we identified adult patients (aged ≥18 years) with a preexisting diagnosis of malignant neoplasm who had both SCr and cystatin C measurements on the same day between May 2010 and January 2022 at 2 major academic cancer centers in Boston, Massachusetts. The eGFRcr was calculated using the Chronic Kidney Disease Epidemiology Collaboration (CKD-EPI) 2021 race-free equation,^5^ eGFRcys was calculated using the CKD-EPI 2012 race-free equation, and eGFRcr-cys was calculated using the CKD-EPI 2021 race-free combined (SCr and cystatin C) equation.^5,17,18,19^

The date of the first simultaneous eGFRcr and eGFRcys measurement was considered to be the baseline date. Comorbidities were defined based on the diagnosis codes used any time prior to the baseline date. Cancer type was ascertained from the most frequent cancer-related diagnosis codes used prior to the baseline date. Medications were identified by the active prescriptions within 1 year prior to the baseline date. Baseline chronic kidney disease was defined by the CKD-EPI 2021 race-free combined equation^7^ and was staged according to the KDIGO (Kidney Disease: Improving Global Outcomes) guidelines.^20^ Clinical diagnoses of medication-related AEs were determined from manual medical record review by 2 investigators (Q.W. and M.E.S.), with a third investigator (P.E.H.) available to resolve disagreement.

### Exposure and Outcomes

The primary exposure was eGFR discordance, defined as an eGFRcys that was more than 30% lower than the eGFRcr, and the reference group consisted of patients with concordant eGFR, defined as eGFRcys that was within 30% of their eGFRcr. We also evaluated the characteristics and risk of medication-related AEs in patients whose eGFRcys was more than 30% higher than their eGFRcr; our a priori hypothesis was that this group would not be at higher risk of medication-related AEs compared with the concordant eGFR group. The 30% cutoff was chosen because it is commonly used in clinical studies to define the accuracy of eGFR from mGFR.^12,21^

For the primary outcome, we examined the risk of selected medication-related AEs using detailed medical record review. We selected medications (intravenous vancomycin, trimethoprim-sulfamethoxazole, baclofen, and digoxin) that are typically dose adjusted based on eGFR and whose adverse effects could be quantified by therapeutic drug monitoring or laboratory abnormalities or identified by medical record review. In all cases, we evaluated drug exposures that occurred within 90 days of the baseline date.

The therapeutic range for a vancomycin trough level was 15 to 20 μg/mL, and levels greater than 20 μg/mL (to convert to micromoles per liter, multiply by 0.69) were considered to be supratherapeutic.^22,23^ We defined severely elevated vancomycin trough levels as greater than 30 μg/mL and used manual medical record review to exclude peak values.^24,25,26^

Trimethoprim-sulfamethoxazole–related hyperkalemia was defined as a serum potassium level greater than 5.5 mEq/L (to convert to millimoles per liter, multiply by 1; Common Terminology Criteria for Adverse Events [CTCAE], version 5.0, grade 2), and severe hyperkalemia was defined as a serum potassium level greater than 6.0 mEq/L (CTCAE grade 3) within 30 days of starting trimethoprim-sulfamethoxazole. In a sensitivity analysis, we identified the mean increase in potassium level after initiation of trimethoprim-sulfamethoxazole.

Baclofen toxic effects were determined from medical record review. Baclofen toxic effects included altered mental status, myoclonus, seizure, or orthostatic hypotension or dizziness warranting discontinuation of the medication.^27,28^ An investigator who was blinded to cystatin C values (Q.W.) evaluated all clinical documentation within 90 days of the baseline date to identify potential cases of baclofen toxic effects.

Digoxin toxic effects were also determined from medical record review. An investigator who was blinded to cystatin C values (Q.W.) evaluated all clinical documentation and digoxin levels obtained within 90 days of the baseline date. We excluded levels that were measured within 6 hours of the previous medication dose. Digoxin toxic effects included altered mental status, nausea, orthostatic hypotension, or bradycardia attributed to digoxin by the treating team, with a corresponding digoxin trough level that was higher than the therapeutic range (>2.0 ng/mL; to convert to nanomoles per liter, multiply by 1.281).^29,30^

For the secondary outcome, we evaluated eGFR discordance (in each direction) as dependent variables and ascertained which baseline characteristics and laboratory studies were associated with eGFR discordance. We also evaluated the 30-day mortality associated with eGFR discordance.

### Statistical Analysis

We reported baseline characteristics using counts and percentages for categorical variables, means and SDs for normally distributed continuous variables, and medians and IQRs for skewed variables. Logistic regression models were used to examine the association among baseline demographic characteristics, comorbidities, medications, laboratory studies, and eGFR discordance in a univariable model. Serum albumin and hemoglobin levels were evaluated in clinically relevant categories shown in the Table. Variables were selected based on clinical plausibility and Akaike and Bayes information criteria to generate the final multivariable model. The Wald χ^2^ test was used to assess the significance of explanatory variables. The final model for an eGFRcys that was more than 30% lower than the eGFRcr was adjusted for age, sex, race and ethnicity (including African American or Black, Asian, Hispanic or Latino, White, or other [Alaskan Indian or American Indian, Hawaiian Native, declined to answer, and not recorded]), eGFRcr-cys, body mass index (BMI), smoking, hypertension, coronary artery disease, diabetes, cirrhosis, malnutrition, thyroid disease, proton pump inhibitor use, diuretic use, corticosteroid use, serum albumin level, and hemoglobin level. Race and ethnicity were evaluated because of their association with genetic risk factors (eg, *Apolipoprotein L1* gene in kidney disease) and health disparities (which are factors in structural racism). The final model for an eGFRcys that was more than 30% higher than the eGFRcr was adjusted for age, sex, race and ethnicity, eGFRcr-cys, BMI, smoking, hypertension, coronary artery disease, diabetes, HIV infection, angiotensin-converting enzyme inhibitor or angiotensin receptor blocker use, proton pump inhibitor use, diuretic use, and corticosteroid use.

**Table.** Patient Characteristics

Characteristic	Patients, No. (%)			
	Overall	eGFRcys >30% lower than eGFRcr	Concordant eGFR	eGFRcys >30% higher than eGFRcr
No. of patients	1869	543	1131	195
Age, mean (SD), y	66 (14)	68 (14)	67 (14)	60 (13)
Sex				
Female	921 (49)	277 (51)	544 (48)	100 (51)
Male	948 (51)	266 (49)	587 (52)	95 (49)
Race and ethnicity^a^				
African American or Black	159 (9)	38 (7)	89 (8)	32 (16)
Asian	67 (4)	19 (4)	38 (3)	10 (5)
Hispanic or Latino	78 (4)	22 (4)	49 (4)	7 (4)
White	1486 (80)	444 (82)	904 (80)	138 (71)
Other^b^	79 (4)	20 (4)	51 (5)	8 (4)
BMI^c^				
Underweight: <18.5	53 (3)	25 (5)	25 (2)	3 (2)
Healthy weight: 18.5-24.9	427 (23)	120 (22)	256 (23)	51 (26)
Overweight: 25-29.9	927 (50)	262 (48)	562 (50)	103 (53)
Obese: ≥30	462 (25)	136 (25)	288 (26)	38 (20)
Smoking	769 (41)	254 (47)	459 (41)	56 (29)
Comorbidities				
Hypertension	1506 (81)	476 (88)	902 (80)	128 (66)
CAD	927 (50)	352 (65)	508 (45)	67 (34)
Diabetes	936 (50)	366 (67)	514 (45)	56 (28)
Cirrhosis	100 (5)	57 (11)	40 (4)	3 (2)
HIV infection	78 (4)	19 (4)	45 (4)	14 (7)
Malnutrition	256 (14)	90 (17)	146 (13)	20 (10)
Thyroid disease	485 (26)	169 (31)	266 (24)	50 (26)
CKD^d^				
eGFR: <30 mL/min/1.73m^2^	483 (47)	196 (51)	249 (44)	38 (54)
eGFR: 30-50 mL/min/1.73m^2^	540 (53)	190 (49)	318 (56)	32 (46)
Medication use				
ACEI or ARB	1053 (56)	320 (59)	650 (58)	83 (43)
Proton pump inhibitors	1219 (65)	411 (76)	711 (63)	97 (50)
Diuretics	1209 (65)	444 (82)	679 (60)	86 (44)
Corticosteroids	377 (20)	197 (36)	161 (14)	19 (10)
Laboratory study, median (IQR)				
Serum creatinine, mg/dL	1.36 (1.02-1.84)	1.29 (0.92-1.78)	1.38 (1.05-1.84)	1.43 (1.17-2.16)
Serum cystatin C, mg/L	1.54 (1.12-2.20)	2.08 (1.60-2.90)	1.39 (1.08-1.92)	0.98 (0.84-1.52)
eGFRcr-cys, mL/min/1.73m^2^	46 (29-68)	37 (25-54)	50 (33-70)	66 (35-82)
Serum albumin, g/dL^c^				
<3.0	281 (15)	185 (34)	80 (7)	16 (8)
3.0-3.99	436 (23)	192 (35)	217 (19)	27 (14)
≥4.0	1152 (62)	166 (31)	834 (74)	152 (78)
BUN, mg/dL^c^				
<17	472 (25)	91 (17)	318 (28)	63 (32)
17-25	499 (27)	103 (19)	334 (30)	62 (32)
>25-39	448 (24)	138 (25)	275 (24)	35 (18)
>39	450 (24)	211 (39)	204 (18)	35 (18)
Hemoglobin, g/dL^c^				
<10.0	541 (29)	289 (53)	214 (19)	38 (20)
10.0-11.9	497 (27)	142 (26)	295 (26)	60 (31)
≥12.0	831 (45)	112 (21)	622 (55)	97 (50)

Abbreviations: ACEI, angiotensin-converting enzyme inhibitor; ARB, angiotensin receptor blocker; BMI, body mass index (calculated as weight in kilograms divided by height in meters squared); BUN, blood urea nitrogen; CAD, coronary artery disease; CKD, chronic kidney disease; eGFR, estimated glomerular filtration rate; eGFRcr, serum creatinine–based eGFR; eGFRcr-cys, eGFR using the Chronic Kidney Disease Epidemiology Collaboration race-free combined (serum creatinine and cystatin C) equation; eGFRcys, cystatin C–based eGFR.

SI conversion factor: To convert BUN to millimoles per liter, multiply by 0.357; hemoglobin to grams per liter, multiply by 10.0; serum albumin to grams per liter, multiply by 10; and serum creatinine to micromoles per liter, multiply by 88.4.

^a^ Race and ethnicity were self-identified by patients and obtained from Mass General Brigham’s centralized data warehouse.

^b^ Other included Alaskan Indian or American Indian, Hawaiian Native, declined to answer, and not recorded.

^c^ Data were missing for the following: BMI was missing for 433 participants (23%), serum albumin level was missing for 62 participants (3%), hemoglobin level was missing for 43 participants (2%), and BUN level was missing for 1 participant (0.05%). The remaining data were complete.

^d^ Chronic kidney disease was staged using the eGFRcr-cys.

The χ^2^ or Fisher exact tests were used to assess differences in the unadjusted rate of medication-related AEs across groups, as appropriate. As a sensitivity analysis, we performed univariable and multivariable logistic regressions to estimate the odds of an elevated vancomycin level greater than 30 μg/mL; the final multivariable model was adjusted for age, sex, race and ethnicity, baseline eGFRcr-cys, BMI, diabetes, corticosteroid use, and hemoglobin level. All comparisons were 2-sided, with *P* < .05 considered to be statistically significant.

Kaplan-Meier survival curves and multivariable Cox proportional hazards regression models were used to compare 30-day survival across groups. The final multivariable model was adjusted for age, sex, race and ethnicity, baseline eGFRcr-cys, BMI, coronary artery disease, diabetes, cirrhosis, malnutrition, angiotensin-converting enzyme inhibitor or angiotensin receptor blocker use, proton pump inhibitor use, diuretic use, corticosteroid use, serum albumin level, and hemoglobin level. The date of the simultaneous assessment of eGFRcr and eGFRcys served as day 0. Patients who were lost to follow-up within 30 days were censored at their last visit. All analyses were performed using R, version 4.1.1 (R Foundation for Statistical Computing); SAS, version 9.4 (SAS Institute Inc), and GraphPad PRISM, version 9.1.0 (GraphPad Software).

## Results

### Baseline Characteristics

There were 1869 patients with cancer who had a simultaneous SCr and cystatin C measurement between May 2010 and January 2022 (eFigure 1 in Supplement 1). These patients had a mean (SD) age of 66 (14) years and included 921 females (49%) and 948 males (51%), most of whom (1486 [80%]) identified as non-Hispanic White (Table). Patients with a wide array of cancer types were included (eTable 1 and eTable 2 in Supplement 1).

**Figure 1.**
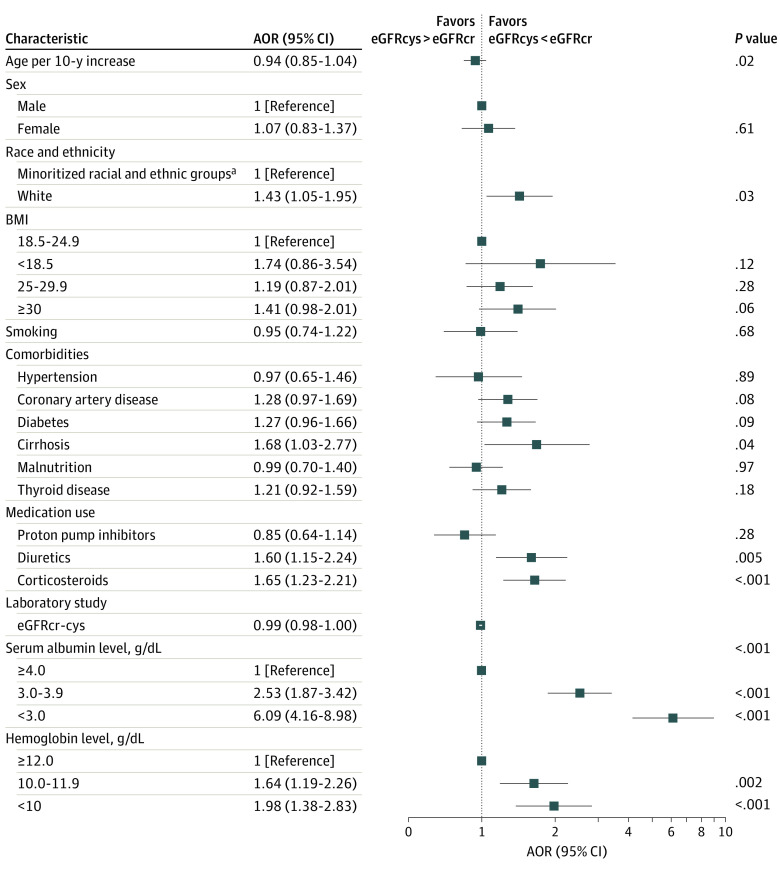
Factors in Estimated Glomerular Filtration Rate (eGFR) Discordance Logistic regression models were used to estimate the association between baseline characteristics and having a cystatin C–based eGFR (eGFRcys) that was more than 30% lower than the serum creatinine–based eGFR (eGFRcr) or an eGFRcys that was more than 30% higher than the eGFRcr. AOR indicates adjusted odds ratio; BMI, body mass index (calculated as weight in kilograms divided by height in meters squared); eGFRcr-cys, eGFR using the Chronic Kidney Disease Epidemiology Collaboration race-free combined equation. To convert hemoglobin level to grams per liter, multiply by 10.0; serum albumin level to grams per liter, multiply by 10. ^a^Race and ethnicity were self-identified. Minoritized racial and ethnic groups included African American or Black, Asian, Hispanic or Latino, and other (including Alaskan Indian or American Indian, Hawaiian Native, declined to answer, and not recorded).

A total of 543 patients (29%) had an eGFRcys that was more than 30% lower than the eGFRcr. A scatterplot of eGFRcr vs eGFRcys is provided in eFigure 2A in Supplement 1, and the distribution of the differences between eGFRcr and eGFRcys is shown in eFigure 2B in Supplement 1. The reference group included patients with concordant eGFR. Factors associated with an eGFRcys that was more than 30% lower than the eGFRcr in the multivariable logistic model included White race (adjusted odds ratio [AOR], 1.43; 95% CI, 1.05-1.95; *P* = .03), cirrhosis (AOR, 1.68; 95% CI, 1.03-2.77; *P* = .04), diuretic use (AOR, 1.60; 95% CI, 1.15-2.24; *P* = .005), recent corticosteroid use (AOR, 1.65; 95% CI, 1.23-2.21; *P* < .001), hypoalbuminemia (serum albumin level <3.0 g/dL [to convert to grams per liter, multiply by 10]: AOR, 6.09; 95% CI, 4.16-8.98; *P* < .001), anemia (hemoglobin level <10.0 g/dL [to convert to grams per liter, multiply by 10]: AOR, 1.98; 95% CI, 1.38-2.83; *P* < .001), and eGFRcr-cys (AOR, 0.99; 95% CI, 0.98-1.00; *P* < .001) (Figure 1; eTable 3 in Supplement 1).

**Figure 2.**
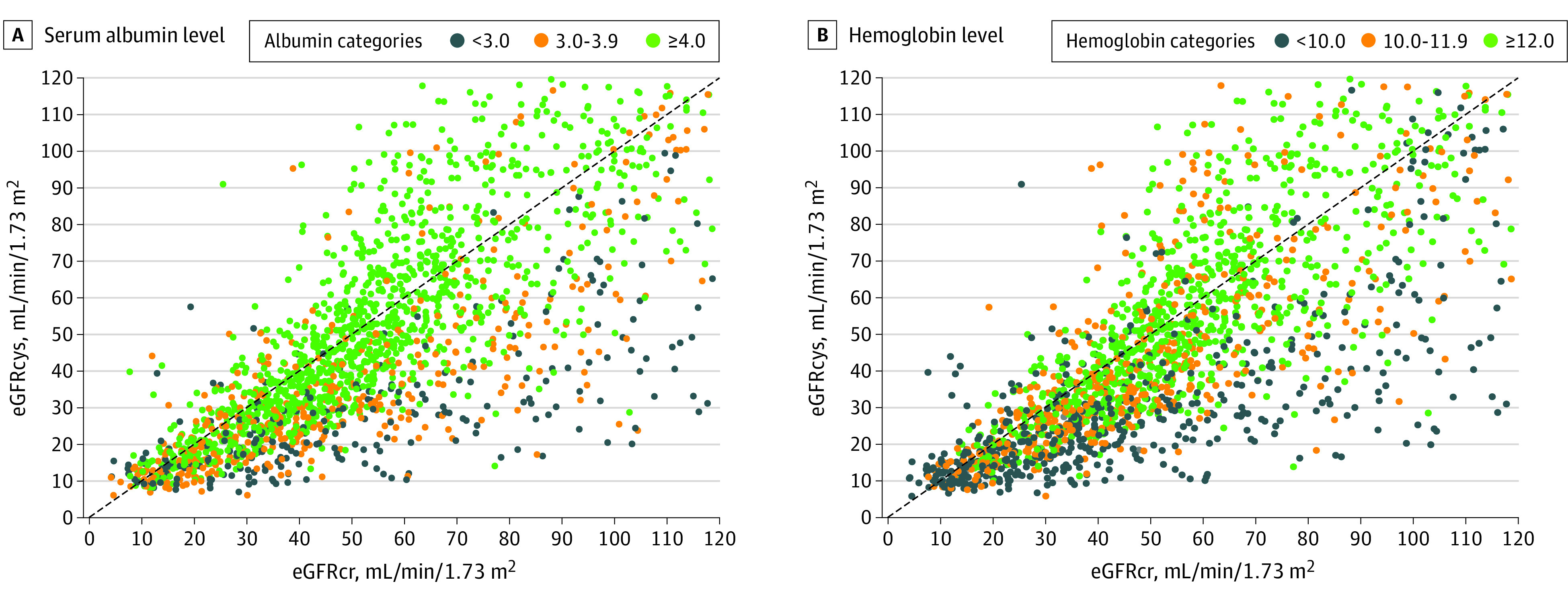
Scatterplots of Serum Creatinine–Based Estimated Glomerular Filtration Rate (eGFRcr) and Cystatin C–Based eGFR (eGFRcys) by Serum Albumin and Hemoglobin Levels The identity line (line of equality) is shown in black.

Hypoalbuminemia and anemia were the baseline factors most associated with an eGFRcys that was more than 30% lower than the eGFRcr (Figure 1). Figure 2 is a scatterplot of eGFRcr vs eGFRcys by serum albumin and hemoglobin levels.

There were 195 patients (10% of the cohort) whose eGFRcys was 30% higher than the eGFRcr. As shown in the Table, patients whose eGFRcys was more than 30% higher than eGFRcr were younger, had fewer medical comorbidities, were less likely to smoke, and had higher baseline eGFRcr-cys than patients with concordant eGFR. HIV infection was more common in patients with an eGFRcys that was more than 30% higher than the eGFRcr due to the frequent use of antiretroviral medications that inhibit creatinine secretion (eg, dolutegravir and cobisctat). Factors associated with an eGFRcys that was more than 30% higher than the eGFRcr in the multivariable logistic model included age (AOR per 10-year increase, 0.75; 95% CI, 0.66-0.86; *P* < .001), White race (AOR, 0.62; 95% CI, 0.43-0.89; *P* = .01), diabetes (AOR, 0.60; 95% CI, 0.40-0.89; *P* = .01), and eGFRcr-cys (AOR, 0.99; 95% CI, 0.99-1.00; *P* = .03) (eFigure 4 and eTable 4 in Supplement 1).

### Medication-Related AEs

**Figure 3.**
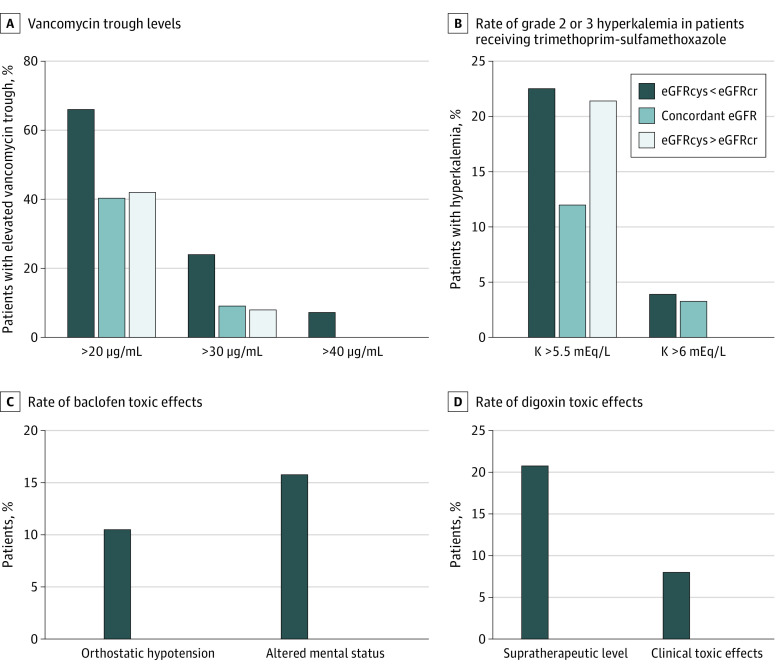
Supratherapeutic Trough Levels and Adverse Events in Patients With a Cystatin C–Based Estimated Glomerular Filtration Rate (eGFRcys) More Than 30% Lower Than the Serum Creatinine–Based eGFR (eGFRcr), Concordant eGFR, or an eGFRcys More Than 30% Higher Than the eGFRcr A, Highest trough levels were obtained within 30 days of starting vancomycin. B. Hyperkalemia was graded using Common Terminology Criteria for Adverse Events, version 5. C, There were no cases of baclofen toxic effects in patients with a concordant eGFR or an eGFRcys more than 30% higher than the eGFRcr. D, Supratherapeutic level was more than 2.0 ng/mL. Both cases of clinical toxic effects occurred in patients with an eGFRcys more than 30% lower than the eGFRcr. There were no cases of supratherapeutic digoxin toxic effects in patients with a concordant eGFR or an eGFRcys more than 30% higher than the eGFRcr.

There were 268 patients who received vancomycin within 90 days of the baseline date and had their vancomycin trough level measured (eFigure 1 in Supplement 1). Patients with an eGFRcys that was more than 30% lower than the eGFRcr were more likely to have significantly elevated vancomycin trough levels than patients with concordant eGFR and patients with an eGFRcys that was more than 30% higher than the eGFRcr (43 of 179 [24%] vs 7 of 77 [9%]; *P* = .01) (Figure 3A). After adjustment for baseline demographic characteristics, comorbidities, and baseline laboratory studies, patients with an eGFRcys that was more than 30% lower than the eGFRcr had a 2.59-fold AOR (95% CI, 1.08-7.03; *P* = .04) of having a significantly elevated vancomycin trough level that was more than 30 μg/mL (eTable 5 in Supplement 1).

There were 235 patients who received trimethoprim-sulfamethoxazole within 90 days of the baseline date and had their serum potassium level checked within 30 days (eFigure 1 in Supplement 1). Grade 2 hyperkalemia was numerically higher in patients with an eGFRcys that was more than 30% lower than the eGFRcr compared with the eGFR-concordant group (29 of 129 [22%] vs 11 of 92 [12%]; *P* = .07) (Figure 3B). A similar pattern was found when evaluating the rate of grade 3 hyperkalemia (defined as potassium level >6.0 mEq). There was also a nonsignificant increase in grade 2 hyperkalemia in patients with an eGFRcys that was more than 30% higher than the eGFRcr (3 of 14 patients [21%]), but there were no episodes of grade 3 hyperkalemia in these patients (Figure 3B). The mean increase in potassium level after initiation of trimethoprim-sulfamethoxazole was higher in patients with an eGFRcys that was more than 30% lower than the eGFRcr compared with patients with concordant eGFR and those with an eGFRcys that was more than 30% higher than the eGFRcr (eFigure 3 in Supplement 1).

Baclofen was newly prescribed to 31 patients within 90 days of the baseline date (eFigure 1 in Supplement 1). Five of the 19 patients (26%) with an eGFRcys that was more than 30% lower than the eGFRcr developed clinical evidence of baclofen toxic effects, which prompted discontinuation of the medication compared with none of the patients with concordant eGFR or with an eGFRcys that was more than 30% higher than the eGFRcr (0 of 11 or 1 of 11; *P* = .19) (Figure 3C). The most common symptom of baclofen toxic effects was altered mental status (3 cases), and 2 other patients developed severe orthostatic hypotension.

Digoxin was newly prescribed to 99 patients (eFigure 1 in Supplement 1), of whom 34 (34%) had at least 1 digoxin level measured. Of the 24 patients with an eGFRcys that was more than 30% lower than the eGFRcr, 7 (29%) had a digoxin trough level greater than the therapeutic range (>2.0 ng/mL) compared with 0 of the 10 patients in the eGFR-concordant group (*P* = .08). Two patients (8.3%) with an eGFRcys that was more than 30% lower than the eGFRcr were diagnosed with clinical digoxin toxic effects, including 1 who required digoxin immune fab (eTable 6 in Supplement 1).

### 30-Day Survival

**Figure 4.**
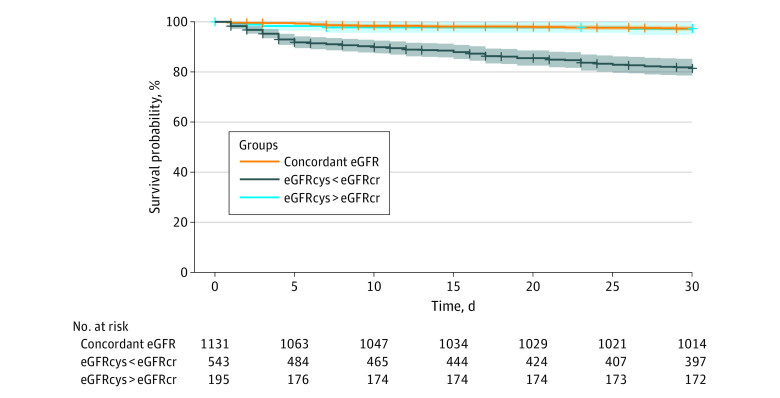
Kaplan-Meier Curve of 30-Day Survival in Patients With a Cystatin C–Based Estimated Glomerular Filtration Rate (eGFRcys) More Than 30% Lower Than the Serum Creatinine–Based eGFR (eGFRcr), Concordant eGFR, or an eGFRcys More Than 30% Higher Than the eGFRcr Unadjusted and adjusted models are provided in eTable 7 in Supplement 1. Shaded areas represent 95% CIs.

One hundred twenty-six patients (7%) died within 30 days, and 160 patients (9%) were lost to follow-up prior to 30 days. There was a significantly higher 30-day mortality in patients with an eGFRcys that was more than 30% lower than the eGFRcr compared with patients with concordant eGFR (Figure 4). Even after adjustment for age, sex, race and ethnicity, baseline comorbidities, laboratory studies, and medication use, patients with an eGFRcys that was more than 30% lower than the eGFRcr had a 1.98-fold increased hazard of death within 30 days (95% CI, 1.26-3.11; *P* = .003) (eTable 3 in Supplement 1). Patients whose eGFRcys was more than 30% higher than the eGFRcr were not at increased risk of death compared with patients with concordant eGFR (Figure 4; eTable 7 in Supplement 1).

## Discussion

In a cohort of 1869 patients with cancer with simultaneous SCr and cystatin C measurement as a part of clinical care, discordancies between eGFRcr and eGFRcys were common. We found a considerably higher rate of supratherapeutic drug levels and AEs associated with select renally cleared medications and increased risk of death in patients with an eGFRcys that was more than 30% lower than the eGFRcr compared with patients with a concordant eGFR or those whose eGFRcys was more than 30% higher than the eGFRcr.

This study found a higher rate of supratherapeutic vancomycin levels in patients with an eGFRcys that was more than 30% lower than the eGFRcr. Several studies have demonstrated that vancomycin clearance and target trough achievement are more accurately associated with eGFRcys than eGFRcr,^31,32,33,34,35^ including 1 study that found that a vancomycin dosing algorithm using eGFRcr-cys (n = 135) was more likely to achieve therapeutic vancomycin trough levels (50% vs 28%; *P* < .001) compared with an algorithm using eGFRcr alone (n = 264).^33^ Trimethoprim-sulfamethoxazole is another commonly used antibiotic in patients with cancer. By inhibiting tubular secretion of potassium, patients with a reduced eGFR are more likely to develop hyperkalemia when treated with trimethoprim-sulfamethoxazole.^36^ In the present study, hyperkalemia occurred more commonly in patients with eGFR discordance. Digoxin is a renally cleared medication used in congestive heart failure or atrial fibrillation that has narrow therapeutic index and dose-dependent toxic effects. There have been conflicting reports regarding the use of SCr vs cystatin C to estimate digoxin clearance.^37,38,39,40,41^ Herein, we found that patients with an eGFRcys that was more than 30% lower than the eGFRcr were more likely to have supratherapeutic digoxin trough levels, and 2 patients had symptomatic digoxin toxic effects. Baclofen is a renally cleared muscle relaxant that is used in patients with cancer and may play a role in profound central nervous system suppression in patients with reduced GFR.^42,43^ The present study showed that symptomatic baclofen toxic effects were common in patients with an eGFRcys that was more than 30% lower than the eGFRcr, whereas no events occurred in patients with a concordant eGFR or those with an eGFRcys that was more than 30% higher than the eGFRcr. Overall, the findings suggest that relying on SCr-based eGFR alone for medication dosing may be inadequate in patients with cancer and highlight the need to consider using eGFRcr-cys when the eGFRcys was more than 30% lower than the eGFRcr.

Consistent with prior knowledge that incorporation of cystatin C adds precision to the eGFR equation in patients with malnutrition, sarcopenia, and cirrhosis,^12,44,45^ we found that hypoalbuminemia and anemia had an important role in detecting an eGFRcys that was more than 30% lower than the eGFRcr in patients with cancer. Additionally, we found that eGFR discordance was associated with a significantly higher 30-day mortality for patients with an eGFRcys that was more than 30% lower than the eGFRcr after adjusting for demographic characteristics, comorbidities, baseline laboratory studies, and medication use. There was no difference in mortality between patients with a concordant eGFR and patients whose eGFRcys was more than 30% higher than the eGFRcr. This finding suggests that having an eGFR discordance, in addition to absolute eGFR values, adds further prognostic information.^46,47,48,49,50,51^

In 2021, the National Kidney Foundation and the American Society of Nephrology Task Force recommended that clinicians estimate GFR using an equation that incorporates both cystatin C and SCr assessments in patients with or at risk for kidney disease.^21^ This combined equation may be particularly important in patients with cancer who may have large discordance between eGFRcr and eGFRcys. An analysis of the accuracy of eGFRcr and eGFRcys in a population of 1200 patients with solid tumors found that in numerous subgroups (eg, those with metastatic disease, low performance status, hypoalbuminemia, and elevated C-reactive protein levels), eGFRcys underestimated mGFR, eGFRcr overestimated mGFR, and the combined equation was the most accurate.^12^ A recent population study with mGFR in more than 6000 patients confirmed that among patients with discordant eGFRcys and eGFRcr, the combined equation was still the most accurate way to estimate GFR.^52^ Because we lacked mGFR, we were unable to assess the accuracy of eGFRcr and eGFRcys; however, we found that when there was a large eGFR discordance, patients were at a higher risk of renally cleared medication–related events and increased risk of death. In this study, we did not find an increased risk of medication-related AEs or increased risk of death in patients whose eGFRcys was more than 30% higher than the eGFRcr.

The findings were in line with results of studies suggesting that an eGFRcys that was lower than the eGFRcr was associated with adverse clinical outcomes.^53,54,55,56^ Among more than 4000 adults with chronic kidney disease who were enrolled in the Chronic Renal Insufficiency Cohort study, eGFRdiff (defined as eGFRcys minus eGFRcr) less than –15 mL/min/1.73m^2^ was associated with increased risk of incident heart failure hospitalization,^53^ end-stage kidney disease, and death.^54^ Two large studies showed that a lower eGFRdiff was associated with higher risk of frailty.^55,56^ In patients with cancer, whose discrepancies in eGFRcr and eGFRcys have been described,^52,57^ the association of eGFR discordance with adverse clinical outcomes has not been reported.

### Limitations

This study has several limitations. First, cystatin C data were only available for select patients for whom a cystatin C test was ordered as a part of routine care. Since cystatin C was not routinely checked in clinical practice, cystatin C levels in the study population were likely enriched for patients with an eGFR discrepancy or potential kidney injury. Over the 12-year study period, cystatin C was measured in only 1869 patients with cancer, whereas more than 770 000 patients with cancer had their SCr checked (approximately 0.2% of the population had concurrent eGFRcr and eGFRcys) over the same period (eFigure 1 in Supplement 1). Thus, the present study almost certainly overestimated the rate of eGFR discordance in the population with cancer in general; however, the selection bias should be balanced between the discordant and concordant eGFR groups, which preserves the validity of comparison of medication-related AEs between the 2 groups. Second, we used a 1-time assessment of SCr and cystatin C, which may not reflect a steady state at the time of measurement.

Third, we were not able to identify cancer stage and measures of functional status from the electronic health record, each of which may be important non-GFR factors in cystatin C and SCr.^58,59^ Fourth, clinician knowledge of the eGFRcys could have altered medication dosing; however, such practice would have biased the results toward the null. Accordingly, the magnitude of association between eGFR discordance and medication-related AEs was likely underestimated. Fifth, elevated trough levels alone of vancomycin and digoxin were not AEs; however, they were important risk factors for developing AEs.^60,61,62,63^ Furthermore, validation of therapeutic drug monitoring levels have not been performed in a population with cancer and could be different due to the unique characteristics of this population that may affect drug pharmacokinetics. Sixth, the study lacked the criterion standard GFR measurement given its retrospective design; however, comparing the adverse outcomes of renally cleared medications served as a surrogate for accuracy of the eGFR.

## Conclusion

In this cohort study, we found that an eGFRcys that was more than 30% lower than the eGFRcr was associated with increased renally cleared medication–related adverse events. Future prospective studies are needed to improve and personalize the approach to GFR estimation and medication dosing in patients with cancer.

## Notes

Supplement 1. **eFigure 1.** Patient Flow **eFigure 2.** Scatter Plot of eGFR_CRE_ and eGFR_CYS_, and Distribution of eGFR Difference **eFigure 3.** Changes in Potassium Levels Among Patients Who Received Trimethoprim-Sulfamethoxazole **eTable 1.** Cancer Types **eTable 2.** ICD 9/10 Codes for Cancer Diagnoses **eTable 3.** Predictors of eGFR_CYS_ < eGFR_CRE_ **eFigure 4. **eGFR_CYS_ >30% Higher Than eGFR_CRE_ **eTable 4.** Predictors of eGFR_CYS_ > eGFR_CRE_ **eTable 5.** Predictors of Vancomycin Level > 30 g/dL **eTable 6.** Deidentified Case Summaries of Clinical Digoxin Toxicity **eTable 7.** Predictors of 30-Day Mortality

Supplement 2.
Data Sharing Statement
