# Supplementary material for: Medication-Related Adverse Events and Discordancies in Cystatin C–Based vs Serum Creatinine–Based Estimated Glomerular Filtration Rate in Patients With Cancer
Source: JAMA Netw Open. 2023 Jul 5;6(7):e2321715. doi: 10.1001/jamanetworkopen.2023.21715 (PMC10323710; doi:10.1001/jamanetworkopen.2023.21715)
Supplement: Supplement 1. — eFigure 1. Patient Flow eFigure 2. Scatter Plot of eGFRCRE and eGFRCYS, and Distribution of eGFR Difference eFigure 3. Changes in Potassium Levels Among Patients Who Received Trimethoprim-Sulfamethoxazole eTable 1. Cancer Types eTable 2. ICD 9/10 Codes for Cancer Diagnoses eTable 3. Predictors of eGFRCYS < eGFRCRE eFigure 4. eGFRCYS >30% Higher Than eGFRCRE eTable 4. Predictors of eGFRCYS > eGFRCRE eTable 5. Predictors of Vancomycin Level > 30 g/dL eTable 6. Deidentified Case Summaries of Clinical Digoxin Toxicity eTable 7. Predictors of 30-Day Mortality [file jamanetwopen-e2321715-s001.pdf]

Supplemental Online Content

Hanna PE, Wang Q, Strohbehn IA, et al. Medication-related adverse events and discordancies in cystatin C–based vs serum creatinine–based estimated glomerular filtration rate in patients with cancer. *JAMA Netw Open*. 2023;6(7):e2321715. doi:10.1001/jamanetworkopen.2023.21715

eFigure 1. Patient Flow

eFigure 2. Scatter Plot of $eGFR_{CRE}$ and $eGFR_{CYS}$, and Distribution of eGFR Difference

eFigure 3. Changes in Potassium Levels Among Patients Who Received Trimethoprim-Sulfamethoxazole

eTable 1. Cancer Types

eTable 2. ICD 9/10 Codes for Cancer Diagnoses

eTable 3. Predictors of $eGFR_{CYS} < eGFR_{CRE}$

eFigure 4. $eGFR_{CYS}$ >30% Higher Than $eGFR_{CRE}$

eTable 4. Predictors of $eGFR_{CYS} > eGFR_{CRE}$

eTable 5. Predictors of Vancomycin Level > 30 g/dL

eTable 6. Deidentified Case Summaries of Clinical Digoxin Toxicity

eTable 7. Predictors of 30-Day Mortality

This supplemental material has been provided by the authors to give readers additional information about their work.

© 2023 Hanna PE et al. *JAMA Network Open.*

eFigure 1. Patient flow

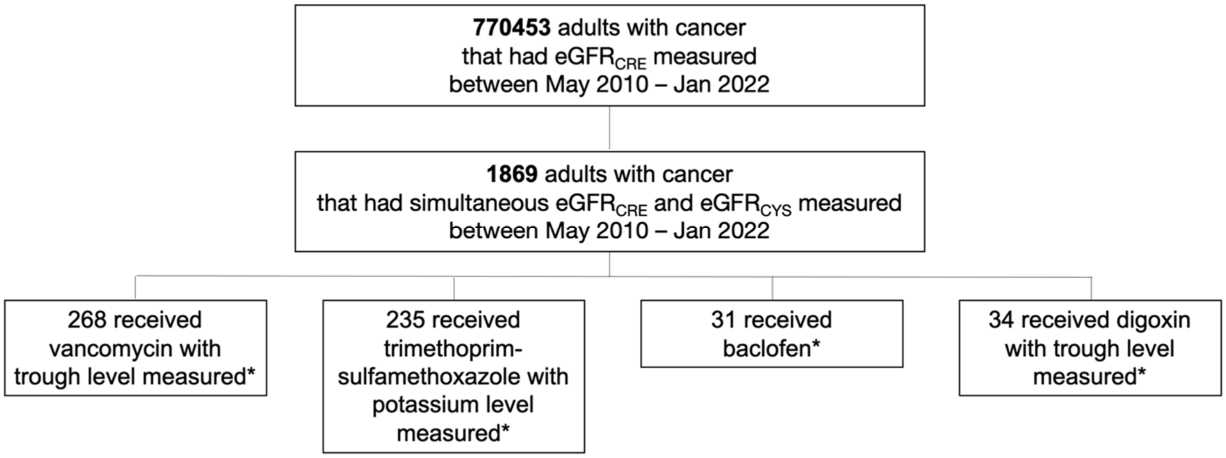

eFigure 1. Patient flow. Exposure to each medication was determined by an active prescription within 90 days of the baseline date. *Shows the analyzed sample for each medication. Abbreviations: $eGFR_{CRE}$ = creatinine-based estimated glomerular filtration rate, $eGFR_{CYS}$ = cystatin c-based estimated glomerular filtration rate.

© 2023 Hanna PE et al. *JAMA Network Open.*

eFigure 2. Scatter Plot of eGFR$_{CRE}$ and eGFR$_{CYS}$, and Distribution of eGFR Difference

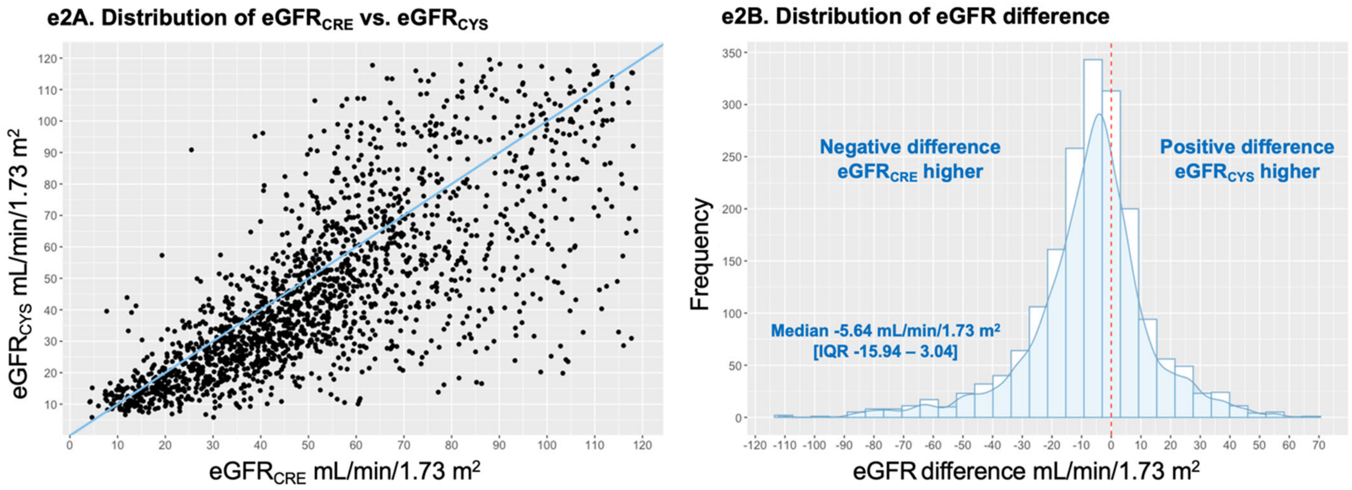

eFigure 2. Scatterplot showing distribution of eGFR$_{CRE}$ and eGFR$_{CYS}$ among patients with cancer (e2A); the blue line is the line of equality. Figure e1B. A histogram and superimposed density curve showing the distribution of eGFR difference defined by eGFR$_{CYS}$ minus eGFR$_{CRE}$ (negative values mean that eGFR$_{CYS}$ is lower than eGFR$_{CRE}$). The red dotted line signifies equivalence between eGFR$_{CRE}$ and eGFR$_{CYS}$. Abbreviations: IQR, interquartile range

© 2023 Hanna PE et al. *JAMA Network Open.*

eFigure 3. Changes in Potassium Levels Among Patients Who Received Trimethoprim-Sulfamethoxazole

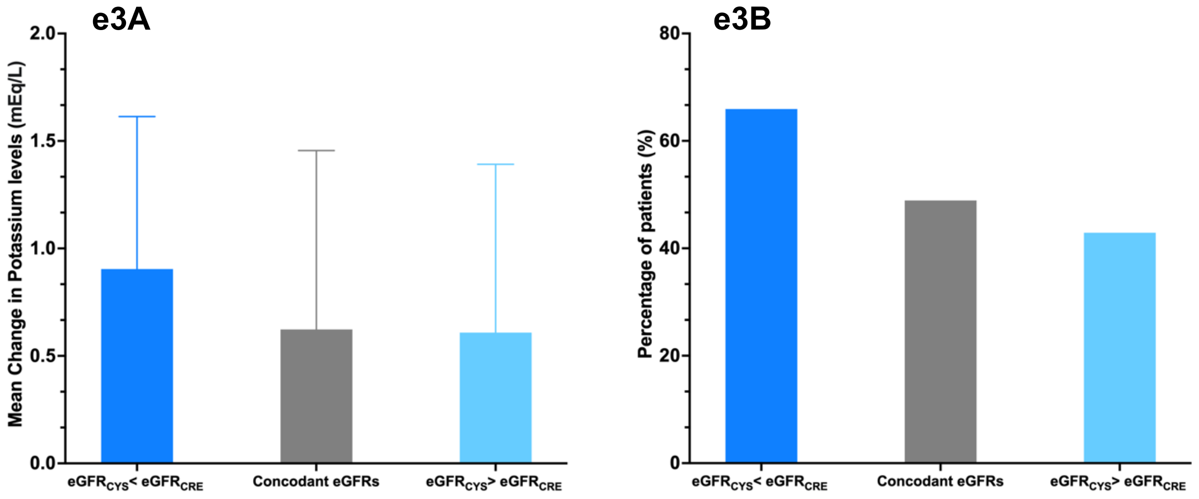

eFigure 3A. Mean change in potassium levels with standard deviations among trimethoprim-sulfamethoxazole recipients; error bars show standard deviation. **eFigure 3B**. Percentage of patients with ≥0.5mEq/L rise in potassium levels after beginning trimethoprim-sulfamethoxazole.

© 2023 Hanna PE et al. *JAMA Network Open.*

eTable 1. Cancer type

	N	eGFR$_{CYS}$< eGFR$_{CRE}$	eGFR$_{CYS}$ ≈ eGFR$_{CRE}$	eGFR$_{CYS}$> eGFR$_{CRE}$	Age (mean (SD))	Male (%)	Female (%)	Cirrhosis (%)	Baseline Albumin (median [IQR])	Baseline Hemoglobin (median [IQR])
**Adrenal**	**4**	2 (50)	1 (25)	1 (25)	62 (17)	4 (100)	0 (0)	1 (25)	4.1 [3.4, 4.6]	12.6 [11.5, 13.1]
**Bladder**	**89**	22 (25)	62 (70)	5 (6)	72(10)	72 (81)	17 (19)	1 (1)	4.1 [3.4, 4.4]	12.1 [9.8, 13.70]
**Brain**	**41**	9 (22)	28 (68)	4 (10)	59 (13)	18 (44)	23 (56)	1 (2)	4.3 [3.9, 4.5]	12.3 [10.7, 14.1]
**Breast**	**227**	37 (16)	153 (68)	37 (16)	63 (15)	4 (2)	223 (98)	8 (4)	4.1 [3.9, 4.4]	11.6 [10.0, 12.7]
**Gastrointestinal**	**202**	73 (36)	112 (55)	17 (8)	69 (13)	123 (61)	79 (39)	24 (12)	4.0 [3.1, 4.4]	11.0 [9.0, 12.8]
**Gynecological**	**146**	37 (25)	95 (65)	14 (10)	64 (12)	5 (3)	141 (97)	5 (3)	4.0 [3.5, 4.4]	11.2 [9.4, 12.7]
**Head and Neck**	**44**	15 (34)	25 (57)	4 (9)	66 (15)	35 (80)	9 (20)	0 (0.0)	4.1 [3.2, 4.6]	11.8 [9.6, 13.3]
**Leukemia**	**131**	66 (50)	58 (44)	7 (5)	63 (14)	86 (66)	45 (34)	13 (10)	3.6 [2.7, 4.3]	9.4 [8.2, 12.2]
**Lymphoma**	**69**	27 (39)	35 (51)	7 (10)	62 (15)	40 (58)	29 (42)	6 (9)	4.0 [2.9, 4.5]	11.6 [8.6, 13.5]
**Melanoma**	**13**	6 (46)	6 (46)	1 (8)	69 (21)	10 (77)	3 (23)	0 (0.0)	4.2 [3.7, 4.5]	12.4 [10.8, 13.9]
**Myeloma**	**62**	25 (40)	31 (50)	6 (10)	67 (10)	26 (42)	36 (58)	1 (2)	4.1 [3.2, 4.3]	11.4 [8.9, 13.1]
**Other cancer**	**74**	25 (34)	38 (51)	11 (15)	62 (15)	34 (46)	40 (54)	6 (8)	4.1 [3.0, 4.4]	11.7 [8.8, 13.1]
**Other Skin cancer**	**343**	84 (25)	221 (64)	38 (11)	68 (15)	205 (60)	138 (40)	21 (6)	4.1 [3.6, 4.5]	12.1 [10.2, 13.9]
**Prostate**	**145**	31 (21)	103 (71)	11 (8)	74 (10)	145 (100)	0 (0)	3 (2)	4.1 [3.9, 4.4]	12.3 [10.7, 14.0]
**Renal**	**114**	28 (25)	74 (65)	12 (11)	60 (15)	76 (67)	38 (33)	2 (2)	4.2 [4.0, 4.6]	12.8 [11.1, 14.1]
**Sarcoma**	**5**	1 (20)	4 (80)	0 (0)	60 (25)	3 (60)	2 (40)	0 (0)	4.3 [4.1, 4.3]	11.7 [11.2, 14.7]
**Testicular**	**1**	0 (0)	1 (100)	0 (0)	40 (NA)	1 (100)	0 (0)	0 (0)	4.5 [4.5, 4.5]	15.2 [15.2, 15.2]
**Thoracic**	**112**	44 (39)	56 (50)	12 (11)	68 (12)	43 (38)	69 (62)	4 (3)	3.9 [3.2, 4.3]	11.1 [9.1, 12.4]
**Thyroid**	**44**	11 (25)	26 (59)	7 (16)	64 (13)	17 (39)	27 (61)	4 (9)	4.1 [3.5, 4.4]	11.8 [10.2, 13.5]
**Ureter**	**3**	0 (0)	2 (67)	1 (33)	62 (16)	1 (33)	2 (67)	0 (0)	4.2 [3.8, 4.6]	12.9 [10.9, 13.5]

eTable 1. Cancer type was determined by the most commonly appearing cancer-related diagnosis code prior to the baseline date.

© 2023 Hanna PE et al. *JAMA Network Open.*

eTable 2. ICD9/10 Codes for Cancer Diagnoses

Adrenal	("194.0", "237.2", "C74.00", "C74.01", "C74.10", "C74.90", "C74.92", "D44.10", "D44.12")
Bladder	("188.0", "188.1", "188.2", "188.3", "188.4", "188.5", "188.8", "188.9", "233.7", "236.7", "239.4", "C23", "C67.0", "C67.1", "C67.2", "C67.3", "C67.4", "C67.5", "C67.8", "C67.9", "D09.0", "D37.6", "D41.4", "D49.4", "V10.51", "Z85.51")
Breast	("174.0", "174.1", "174.2", "174.3", "174.4", "174.5", "174.6", "174.8", "174.9", "175.0", "175.9", "233.0", "238.3", "239.3", "C43.52", "C44.521", "C44.591", "C50.011", "C50.012", "C50.019", "C50.111", "C50.112", "C50.119", "C50.121", "C50.211", "C50.212", "C50.219", "C50.221", "C50.311", "C50.312", "C50.321", "C50.322", "C50.411", "C50.412", "C50.419", "C50.511", "C50.512", "C50.519", "C50.611", "C50.612", "C50.811", "C50.812", "C50.819", "C50.821", "C50.911", "C50.912", "C50.919", "C50.921", "C50.922", "C50.929", "D05.00", "D05.01", "D05.10", "D05.11", "D05.12", "D05.90", "D05.91", "D05.92", "V10.3", "Z85.3", "Z86.000")
Gastrointestinal	("150.5", "150.8", "150.9", "151.4", "151.9", "152.8", "153.1", "153.2", "153.3", "153.6", "153.9", "154.1", "154.3", "154.8", "155.0", "157.0", "157.1", "157.2", "157.8", "157.9", "209.00", "209.17", "230.1", "230.3", "230.6", "235.2", "235.3", "C15.3", "C15.4", "C15.5", "C15.8", "C15.9", "C16.1", "C16.2", "C16.5", "C16.6", "C16.8", "C16.9", "C17.9", "C18.2", "C18.4", "C18.6", "C18.7", "C18.8", "C18.9", "C20", "C21.0", "C22.0", "C22.1", "C22.7", "C22.8", "C23", "C24.0", "C24.9", "C25.0", "C25.1", "C25.2", "C25.3", "C25.4", "C25.7", "C25.8", "C25.9", "C7A.019", "C7A.026", "C7A.092", "D00.1", "D37.1", "D37.4", "D37.5", "D37.6", "V10.00", "V10.03", "V10.04", "V10.06", "V10.09", "Z85.01", "Z85.020", "Z85.028", "Z85.040", "Z85.048", "Z85.05", "Z85.060", "Z85.068", "Z85.07")
Gynecological	("179.0", "180.0", "180.8", "180.9", "182", "182.8", "183.0", "183.5", "183.8", "183.9", "184.0", "184.4", "184.8", "184.9", "233.1", "233.3", "233.32", "236.0", "236.2", "236.3", "C51.9", "C52", "C53.0", "C53.1", "C53.8", "C53.9", "C54.1", "C54.8", "C54.9", "C55", "C56.1", "C56.2", "C56.3", "C56.9", "C57.4", "C57.7", "C57.8", "C57.9", "D06.0", "D06.1", "D06.9", "D07.0", "D07.30", "D39.0", "D39.10", "D39.12", "D39.8", "V10.40", "V10.41", "V10.42", "V10.43", "V10.44", "V71.1", "Z85.40", "Z85.41", "Z85.42", "Z85.43", "Z85.44", "Z86.001")
Head and Neck	("140.1", "140.5", "140.9", "141.0", "141.1", "141.2", "141.3", "141.4", "141.8", "141.9", "142.9", "144.0", "144.9", "145.9", "146.9", "149.0", "149.8", "160.0", "160.9", "161.9", "172.0", "173.0", "173.01", "173.02", "191.2", "230.0", "231.0", "235.0", "235.1", "235.6", "C01", "C02.1", "C02.2", "C02.8", "C02.9", "C03.1", "C06.9", "C10.2", "C10.8", "C10.9", "C11.0", "C11.1", "C11.9", "C12", "C13.1", "C13.9", "C14.0", "C30.0", "C31.0", "C31.3", "C31.8", "C31.9", "C32.9", "C41.1", "C43.0", “C44.01", "C44.02", "C44.301", "C44.311", "C44.321", "C49.0", "C4A.31", "C71.2", "C76.0", "D00.07", "D02.0", "D04.0", "D37.030", "D37.039", "D37.04", "V10.01", "V10.02", "V10.21", "V10.22", "Z85.21", "Z85.22", "Z85.810", "Z85.818", "Z85.819")
Leukemia	("203.10", "204.00", "204.01", "204.02", "204.1", "204.10", "204.11", "204.12", "204.20", "204.80", "204.9", "204.90", "205.0", "205.00", "205.01", "205.02", "205.1", "205.10", "205.11", "205.30", "205.90", "205.91", "205.92", "206.00", "207.0", "207.80", "208.0", "208.00", "208.01", "208.10", "208.8", "208.80", "208.9", "208.90", "208.91", "208.92", "C90.10", "C91.00", "C91.01", "C91.02", "C91.10", "C91.11", "C91.12", "C91.50", "C91.51", "C91.52", "C91.60", "C91.90", "C91.91", "C91.92", "C91.Z0", "C91.Z1", "C91.Z2", "C92.00", "C92.01", "C92.02", "C92.10", "C92.11", "C92.20", "C92.21", "C92.30", "C92.32", "C92.40", "C92.41", "C92.42", "C92.50", "C92.51", "C92.52", "C92.90", "C92.A0", "C92.A1", "C92.A2", "C92.Z1", "C92.Z2",

© 2023 Hanna PE et al. *JAMA Network Open.*

	"C93.00", "C93.01", "C93.10", "C93.11", "C94.31", "C94.80", "C95.00", "C95.01", "C95.02", "C95.90", "C95.92", "V10.60", "V10.61", "V10.62", "V10.63", "Z85.6")
Lymphoma	("202.8", "202.80", "202.81", "202.82", "202.83", "202.85", "202.86", "202.88", "202.90", "204.00", "204.01", "204.02", "204.1", "204.10", "204.11", "204.12", "204.20", "204.80", "204.9", "204.90", "C91.50", "C91.51", "C91.52", "C91.90", "C91.91", "C91.92", "C91.Z0", "C91.Z1", "C91.Z2", "C96.9", "D47.9", "D47.Z9", "V10.61", "Z85.79")
melanoma	("172.0", "172.2", "172.3", "172.4", "172.6", "172.7", "172.8", "190.6", "C43.0", "C43.20", "C43.22", "C43.30", "C43.39", "C43.4", "C43.52", "C43.59", "C43.60", "C43.61", "C43.62", "C43.70", "C43.71", "C43.72", "C43.8", "C43.9", "C69.31", "V10.82", "Z85.820")
meningioma	("191.8", "191.9", "192.1", "237.5", "237.6", "239.6", "C70.1", "C71.7", "C71.8", "C71.9", "D42.0", "D42.1", "D42.9", "D43.0", "D43.2", "D49.6", "V10.85", "Z85.841")
Myeloma	("203.0", "203.00", "203.01", "C90.00", "C90.01", "C90.02")
Other skin	("172.0", "172.2", "172.3", "172.4", "172.6", "172.7", "172.8", "173.0", "173.01", "173.02", "173.1", "173.11", "173.12", "173.2", "173.20", "173.21", "173.22", "173.3", "173.30", "173.31", "173.32", "173.39", "173.4", "173.40", "173.41", "173.42", "173.49", "173.6", "173.60", "173.61", "173.62", "173.69", "173.7", "173.70", "173.71", "173.72", "173.79", "173.8", "173.81", "173.82", "173.89", "173.9", "173.90", "173.91", "173.92", "176.0", "209.31", "209.33", "209.36", "209.75", "232.2", "232.3", "232.4", "232.6","232.7", "232.8", "232.9", "238.2", "239.2", "C43.52", "C43.8", "C43.9", "C44.01", "C44.02", "C44.109", "C44.111", "C44.112", "C44.1122", "C44.119", "C44.1192", "C44.129", "C44.1292", "C44.199", "C44.202", "C44.212", "C44.219", "C44.222", "C44.229", "C44.300", "C44.301", "C44.310", "C44.311", "C44.319", "C44.320", "C44.321", "C44.329", "C44.390", "C44.40", "C44.41", "C44.42", "C44.49", "C44.509", "C44.519", "C44.520", "C44.521", "C44.529", "C44.591", "C44.609", "C44.611", "C44.612", "C44.619", "C44.621", "C44.622", "C44.629", "C44.702", "C44.711", "C44.712", "C44.719", "C44.722", "C44.729", "C44.89", "C44.90", "C44.91", "C44.92", "C44.99", "C46.0", "C4A.30", "C4A.31", "C4A.39", "C4A.4", "C4A.59", "C4A.9", "D04.0", "D04.12", "D04.21", "D04.22", "D04.30", "D04.39", "D04.4", "D04.5", "D04.61", "D04.62", "D04.71", "D04.72", "D04.9", "D48.5", "D49.2", "V10.82", "V10.83", "Z85.820", "Z85.821", "Z85.828", "Z86.007")
Other Cancer	("146.0", "171.9", "186.9", "192.2", "194.3", "233.9", "238.1", "238.7", "238.79", "238.8", "239.5", "239.7", "239.8", "239.9", "789.51", "C08.1", "C40.21", "C41.9", "C48.0", "C49.4", "C49.9", "C68.0", "C68.9", "C71.1", "C76.51", "C80.1", "D44.3", "D47.09", "D48.0", "D48.9", "D49.7", "D49.89", "G89.3", "J91.0", "R18.0", "T88.3XXA" , "V10.05", "V10.79", "V10.89", "V10.90", "Z08", "Z85.038", "Z85.830", "Z85.831", "Z85.858", "Z85.89", "Z85.9", "Z86.008")
Prostate	("185", "185.9", "233.4", "236.5", "C61", "D07.5", "R97.21", "V10.46", "Z85.46")
Renal	("189.1", "236.91", "C65.1", "C65.2", "C65.9", "C7A.093", "D41.00", "D41.11", "D41.12", "D49.511", "D49.512", "D49.519", "V10.52", "V10.53", "Z85.520", "Z85.528", "Z85.53")
Sarcoma	("176.0", "176.9", "200.00", "200.01", "200.02", "200.10", "200.18", "200.80", "201.21", "205.30", "C46.0", "C46.1", "C46.3", "C46.7", "C46.9", "C92.30", "C92.32", "C96.A", "V10.71")
Testicular	("186.9", "C62.90", "D40.10")
Thoracic	("162.3", "162.4", "162.5", "162.8", "162.9", "163.8", "163.9", "231.2", "235.7", "235.8", "235.9", "239.1", "C34.10", "C34.11", "C34.12", "C34.2", "C34.30", "C34.31", "C34.32", "C34.80", "C34.81", "C34.82", "C34.90", "C34.91", "C34.92", "C38.4", "D02.20", "D38.1", "D38.5", "D49.1", "V10.11", "V10.29", "Z85.110", "Z85.118", "Z85.29")
Thyroid	("193", "C73", "V10.87", "Z85.850")
Ureter	("188.6", "189.2", "236.91", "C66.1", "C66.2", "C66.9", "C67.6", "D41.21", "D41.22", "Z85.54")

© 2023 Hanna PE et al. *JAMA Network Open.*

eTable 3. Predictors of $eGFR_{CYS}$ < $eGFR_{CRE}$

Covariates	$eGFR_{CYS}$ < $eGFR_{CRE}$					
	Univariable			Multivariable		
	OR	95% CI	p-value	Adj OR	95% CI	p-value
**Age (per 10 years)**	1.05	0.97, 1.13	0.22	0.94	0.85, 1.04	0.21
**Female Sex**	1.12	0.92, 1.38	0.26	1.07	0.83, 1.37	0.61
**White Race**	1.13	0.87, 1.47	0.37	1.43	1.05, 1.95	**0.025**
**Body mass index, kg/m²**						
**Normal (18.5 - 24.9)**	REF	—	—	—	—	—
**Under weight (<18.5)**	2.13	1.17, 3.88	**0.013**	1.74	0.86, 3.54	0.12
**Overweight (25 - 29.9)**	1.0	0.77, 1.29	0.97	1.19	0.87, 1.62	0.28
**Obese (≥ 30)**	1.01	0.75, 1.36	0.96	1.41	0.98, 2.01	0.06
**Smoking**	1.29	1.05, 1.58	**0.017**	0.95	0.74, 1.22	0.68
**Comorbidities**						
**Hypertension**	1.80	1.35, 2.44	**<0.001**	0.97	0.65, 1.46	0.89
**Coronary Artery Disease**	2.26	1.83, 2.80	**<0.001**	1.28	0.97, 1.69	0.08
**Diabetes Mellitus**	2.48	2.01, 3.08	**<0.001**	1.27	0.96, 1.66	0.09
**Cirrhosis**	3.20	2.11, 4.89	**<0.001**	1.68	1.03, 2.77	**0.041**
**Human Immunodeficiency Virus**	0.88	0.50, 1.49	0.63			
**Malnutrition**	1.34	1.01, 1.78	**0.044**	0.99	0.70, 1.40	0.97
**Thyroid disease**	1.47	1.17, 1.84	**<0.001**	1.21	0.92, 1.59	0.18
**Medication Use***						
**ACEi/ARB**	1.06	0.86, 1.31	0.57			
**Proton Pump Inhibitors**	1.84	1.46, 2.32	**<0.001**	0.85	0.64, 1.14	0.28
**Diuretics**	2.99	2.34, 3.84	**<0.001**	1.60	1.15, 2.24	**0.005**
**Corticosteroids**	3.43	2.70, 4.37	**<0.001**	1.65	1.23, 2.21	**<0.001**
**Labs**						
**$eGFR_{CRE-CYS}$**	0.98	0.98, 0.98	**<0.001**	0.99	0.98, 1.0	**<0.001**
**Albumin (g/dL)**						
**≥4.0**	REF	—	—	—	—	—
**3.0 to <4.0**	4.45	3.45, 5.75	**<0.001**	2.53	1.87, 3.42	**<0.001**
**<3.0**	11.6	8.55, 15.9	**<0.001**	6.09	4.16, 8.98	**<0.001**
**Hemoglobin (g/dL)**						
**≥12.0**	REF	—	—	—	—	—
**10.0 to <12.0**	2.67	2.01, 3.56	**<0.001**	1.64	1.19, 2.26	**0.002**
**<10.0**	7.50	5.75, 9.83	**<0.001**	1.98	1.38, 2.83	**<0.001**

Univariable and multivariable logistic regression model. *Chronic medication use was defined within 1 year prior to baseline; corticosteroid use was defined within 30 days of baseline. In all cases the population median was imputed for missing variables (Body mass index was missing for 479 participants, serum albumin was missing for 72 participants, hemoglobin was missing for 46 participants). Abbreviations $eGFR_{CRE-CYS}$ = estimated Glomerular Filtration Rate calculated using the 2021 race-free combined serum creatinine and cystatin C equation, ACEi/ARB = Angiotensin Converting Enzyme Inhibitor/Angiotensin Receptor Blocker.

© 2023 Hanna PE et al. *JAMA Network Open.*

eFigure 4. $eGFR_{CYS}$ >30% Higher Than $eGFR_{CRE}$

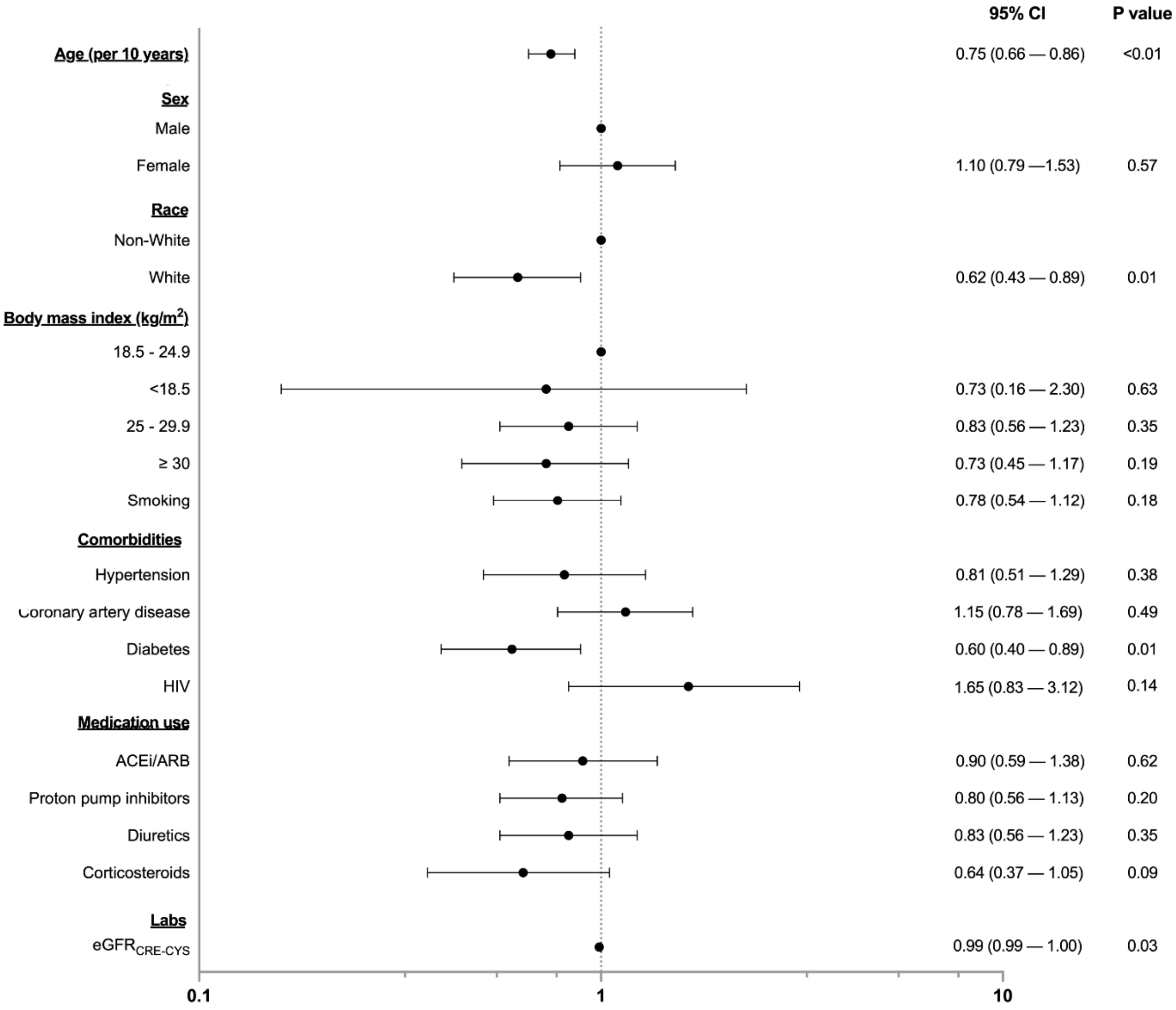

© 2023 Hanna PE et al. *JAMA Network Open.*

eTable 4. Predictors of $eGFR_{CYS}$ > $eGFR_{CRE}$

Covariates	eGFR$_{CYS}$ > eGFR$_{CRE}$					
	Univariable			Multivariable		
	OR	95% CI	p-value	Adj OR	95% CI	p-value
Age (per 10 years)	0.73	0.65, 0.81	**<0.001**	0.75	0.66, 0.86	**<0.001**
Female Sex	1.14	0.84, 1.54	0.41	1.10	0.79, 1.53	0.57
White Race	0.61	0.43, 0.86	**0.004**	0.62	0.43, 0.89	**0.010**
Body mass index, kg/m^2						
Normal (18.5 - 24.9)	REF	—	—	—	—	—
Under weight (<18.5)	0.60	0.14, 1.80	0.42	0.73	0.16, 2.30	0.63
Overweight (25 - 29.9)	0.92	0.64, 1.34	0.66	0.83	0.56, 1.23	0.35
Obese (≥ 30)	0.66	0.42, 1.04	0.07	0.73	0.45, 1.17	0.19
Smoking	0.59	0.42, 0.82	**0.002**	0.78	0.54, 1.12	0.18
Comorbidities						
Hypertension	0.49	0.35, 0.68	**<0.001**	0.81	0.51, 1.29	0.38
Coronary Artery Disease	0.64	0.47, 0.88	**0.006**	1.15	0.78, 1.69	0.49
Diabetes Mellitus	0.48	0.34, 0.67	**<0.001**	0.60	0.40, 0.89	**0.011**
Cirrhosis	0.43	0.10, 1.19	0.16			
Human Immunodeficiency Virus	1.87	0.97, 3.38	**0.049**	1.65	0.83, 3.12	0.14
Malnutrition	0.77	0.46, 1.24	0.30			
Thyroid disease	1.12	0.78, 1.58	0.52			
Medication Use*						
ACEi/ARB	0.55	0.40, 0.74	**<0.001**	0.90	0.59, 1.38	0.62
Proton Pump Inhibitors	0.58	0.43, 0.79	**<0.001**	0.80	0.56, 1.13	0.20
Diuretics	0.53	0.39, 0.71	**<0.001**	0.83	0.56, 1.23	0.35
Corticosteroids	0.65	0.38, 1.05	0.09	0.64	0.37, 1.05	0.09
Labs						
eGFR$_{CRE-CYS}$	1.01	1.00, 1.01	**<0.001**	0.99	0.99, 1.00	**0.032**
Albumin (g/dL)						
≥4.0	REF	—	—	—	—	—
3.0 to <4.0	0.68	0.43, 1.04	0.09			
<3.0	1.10	0.60, 1.88	0.75			
Hemoglobin (g/dL)						
≥12.0	REF	—	—	—	—	—
10.0 to <12.0	1.30	0.92, 1.85	0.14			
<10.0	1.14	0.75, 1.70	0.53			

eTable 4. Univariable and multivariable logistic regression model. *Chronic medication use was defined within 1 year prior to baseline; corticosteroid use was defined within 30 days of baseline. In all cases the population median was imputed for missing variables (Body mass index was missing for 479 participants, serum albumin was missing for 72 participants, hemoglobin was missing for 46 participants). Abbreviations $eGFR_{CRE-CYS}$ = estimated Glomerular Filtration Rate calculated using the 2021 race-free combined serum creatinine and cystatin C equation, ACEi/ARB = Angiotensin Converting Enzyme Inhibitor/Angiotensin Receptor Blocker.

© 2023 Hanna PE et al. *JAMA Network Open.*

eTable 5. Predictors of Vancomycin Level >30μg/dL

Covariates	Predictors of Vancomycin level >30μg/dL(N=51) Vs. <30μg/dL (N=217)					
	Univariable			Multivariable		
	OR	95% CI	p-value	Adj OR	95% CI	p-value
**Age (per 10 years)**	0.8	0.64, 0.98	**0.033**	0.75	0.57, 0.98	**0.036**
**Female Sex**	1.37	0.74, 2.53	0.32	1.49	0.76, 2.95	0.25
**White Race**	1.75	0.74, 4.81	0.23	1.66	0.66, 4.83	0.31
**$eGFR_{CRE}$ vs. $eGFR_{CYS}$**						
**$eGFR_{CYS}$ ~ $eGFR_{CRE}$(within 30)**	—	—		—	—	
**$eGFR_{CYS}$<$eGFR_{CRE}$(<-30)**	3.16	1.43, 8.01	**0.008**	2.59	1.08, 7.03	**0.044**
**$eGFR_{CYS}$>$eGFR_{CRE}$(>30)**	0.91	0.05, 5.86	0.93	0.98	0.05, 7.08	0.98
**Body mass index**	1.04	0.99, 1.10	0.08	1.03	0.98, 1.09	0.21
**Smoking**	0.87	0.47, 1.60	0.65			
**Comorbidities**						
**Hypertension**	1.35	0.57, 3.74	0.53			
**Coronary Artery Disease**	1.21	0.62, 2.44	0.59			
**Diabetes Mellitus**	3.20	1.32, 9.58	**0.019**	2.23	0.83, 7.14	0.14
**Cirrhosis**	1.07	0.41, 2.50	0.88			
**Malnutrition**	0.42	0.10, 1.26	0.17			
**Thyroid disease**	0.85	0.42, 1.64	0.63			
**Medication Use***						
**ACEi/ARB**	0.87	0.47, 1.60	0.65			
**Proton Pump Inhibitors**	1.25	0.52, 3.48	0.64			
**Diuretics**	2.08	0.84, 6.28	0.15			
**Corticosteroids**	3.48	1.80, 7.14	**<0.001**	2.81	1.38, 6.00	**0.005**
**Baseline Labs**						
**Serum Creatinine (mg/dL)**	0.86	0.63, 1.09	0.28			
**$eGFR_{CRE-CYS}$**	0.99	0.98, 1.00	0.06	0.99	0.97, 1.00	0.06
**Baseline Albumin (g/dL)**	0.84	0.51, 1.35	0.47			
**Baseline Hemoglobin (g/dL)**	0.8	0.64, 0.97	**0.032**	0.91	0.71, 1.14	0.43

eTable 5. Univariable and multivariable logistic regression model predicting Vancomycin trough level > 30 μg/dL. Abbreviations: $eGFR_{CRE-CYS}$ = estimated Glomerular Filtration Rate calculated using the 2021 race-free combined serum creatinine and cystatin C equation, ACEi/ARB = Angiotensin Converting Enzyme Inhibitor/Angiotensin Receptor Blocker.

© 2023 Hanna PE et al. *JAMA Network Open.*

eTable 6. Deidentified Case Summaries of Clinical Digoxin Toxicity

65-70-year-old woman with atrial fibrillation and congestive heart failure	Admitted for hypercalcemia due to refractory multiple myeloma. She developed bradycardia with AV block, altered mental status, and hyperkalemia while on digoxin 0.25 mg daily, and her digoxin trough level was 3.8 ng/mL She was treated with digoxin immune fab followed by improvement in bradycardia and mental status. Digoxin was permanently discontinued.
60-65-year-old woman with metastatic neuroendocrine cancer and carcinoid heart disease	Admitted for pulmonic and tricuspid valve replacement surgery and developed recurrent atrial flutter during a prolonged hospital stay. She was treated with digoxin load (0.25mg intravenous for 3 doses) followed by maintenance digoxin 0.125mg oral daily. On hospital day 83, she developed nausea and vomiting attributed to elevated digoxin (trough level was 2.1 ng/mL) and her symptoms fully resolved with discontinuation of digoxin.

© 2023 Hanna PE et al. *JAMA Network Open.*

eTable 7. Predictors of 30-day Mortality

Covariates	30-day mortality					
	Univariable			Multivariable		
	HR	95% CI	p-value	Adj HR	95% CI	p-value
**Age (per 10 years)**	1.01	0.89, 1.15	0.85	1.02	0.88, 1.18	0.77
**Female Sex**	0.72	0.50, 1.02	0.06	0.75	0.52, 1.09	0.13
**White Race**	1.61	0.98, 2.65	0.06	1.55	0.92, 2.60	0.10
**Body mass index**						
**Normal Range**	—	—		—	—	
**Underweight**	1.24	0.48, 3.19	0.65	1.25	0.47, 3.34	0.65
**Overweight**	1.03	0.68, 1.56	0.90	1.05	0.68, 1.62	0.83
**Obese**	0.59	0.34, 1.02	0.06	1.07	0.61, 1.89	0.81
**$eGFR_{CRE}$ vs. $eGFR_{CYS}$**						
**$eGFR_{CYS}$ ~ $eGFR_{CRE}$(within 30)**	—	—		—	—	
**$eGFR_{CYS}$<$eGFR_{CRE}$(<-30)**	7.67	5.03, 11.7	**<0.001**	1.98	1.26, 3.11	**0.003**
**$eGFR_{CYS}$>$eGFR_{CRE}$(>30)**	1.07	0.41, 2.77	0.89	0.91	0.35, 2.42	0.86
**Smoking**	1.18	0.84, 1.68	0.34			
**Comorbidities**						
**Hypertension**	0.83	0.55, 1.26	0.38			
**Coronary Artery Disease**	2.01	1.39, 2.90	**<0.001**	1.01	0.67, 1.53	0.96
**Diabetes Mellitus**	2.42	1.65, 3.55	**<0.001**	1.04	0.67, 1.60	0.88
**Cirrhosis**	2.77	1.66, 4.61	**<0.001**	1.24	0.72, 2.15	0.44
**Human Immunodeficiency Virus**	0.92	0.38, 2.26	0.86			
**Malnutrition**	0.58	0.31, 1.08	0.09	0.52	0.27, 1.00	0.05
**Thyroid disease**	1.23	0.84, 1.79	0.28			
**Medication Use***						
**ACEi/ARB**	0.7	0.49, 0.99	**0.044**	0.88	0.58, 1.31	0.52
**Proton Pump Inhibitors**	2.07	1.34, 3.18	**<0.001**	1.22	0.75, 1.98	0.42
**Diuretics**	1.91	1.26, 2.91	**0.002**	0.72	0.43, 1.20	0.21
**Corticosteroids**	4.76	3.36, 6.73	**<0.001**	1.49	1.01, 2.18	**0.042**
**Baseline Labs**						
**$eGFR_{CRE-CYS}$**	0.98	0.97, 0.99	**<0.001**	0.99	0.98, 1.00	**0.027**
**Albumin (g/dL)**						
**≥4.0**	—	—		—	—	
**3.0 to <4.0**	15.4	5.93, 39.8	**<0.001**	6.71	2.43, 18.5	**<0.01**
**<3.0**	94.3	38.4, 232	**<0.001**	29.7	10.9, 81.0	**<0.01**
**Hemoglobin (g/dL)**						
**≥12.0**	—	—		—	—	
**10.0 to <12.0**	5.79	2.32, 14.4	**<0.001**	1.92	0.74, 5.01	0.18
**<10.0**	28.7	12.6, 65.3	**<0.001**	2.96	1.19, 7.36	**0.020**

*Chronic medication use was defined within 1 year prior to baseline; corticosteroid use was defined within 30 days of baseline. In all cases the population median was imputed for missing variables (body mass index was missing for 479 participants, serum albumin was missing for 72 participants, hemoglobin was missing for 46 participants).

© 2023 Hanna PE et al. *JAMA Network Open.*

Abbreviations: $eGFR_{CRE-CYS}$ = estimated Glomerular Filtration Rate calculated using the 2021 race-free combined serum creatinine and cystatin C equation, ACEi/ARB = Angiotensin Converting Enzyme Inhibitor/Angiotensin Receptor Blocker.

© 2023 Hanna PE et al. *JAMA Network Open.*
