# Supplementary material for: Medication-Related Adverse Events and Discordancies in Cystatin C–Based vs Serum Creatinine–Based Estimated Glomerular Filtration Rate in Patients With Cancer
Source: JAMA Netw Open. 2023 Jul 5;6(7):e2321715. doi: 10.1001/jamanetworkopen.2023.21715 (PMC10323710; doi:10.1001/jamanetworkopen.2023.21715)
Supplement: Supplement 2. — Data Sharing Statement [file jamanetwopen-e2321715-s002.pdf]

Data Sharing Statement

Hanna. Medication-Related Adverse Events and Discordancies in Cystatin C–Based vs Serum Creatinine–Based Estimated Glomerular Filtration Rate in Patients With Cancer. *JAMA Netw Open*. Published July 05, 2023. doi:10.1001/jamanetworkopen.2023.21715

Data
Data available: Yes
Data types: Deidentified participant data
How to access data: Deidentified participant data, data dictionary, will be made available on reasonable written request by email to the corresponding author (msise@partners.org) after execution of data use agreement.
When available: With publication

Supporting Documents
Document types: None

Additional Information
Who can access the data: Deidentified participant data, data dictionary, will be made available on reasonable written request by anyone requesting the data by email to the corresponding author (msise@partners.org) after execution of data use agreement.
Types of analyses: for any purpose
Mechanisms of data availability: with a signed data access agreement
Any additional restrictions: none
